# Virtual reality simulation for facilitating critical reflection and transformative learning: pedagogical, practical, and ethical considerations

**DOI:** 10.1186/s41077-024-00319-x

**Published:** 2024-12-18

**Authors:** Niki Soilis, Farhan Bhanji, Elizabeth Anne Kinsella

**Affiliations:** 1 Institute of Health Sciences Education, Faculty of Medicine & Health Sciences, McGill University, Montreal, QC Canada; 2 Institute of Health Sciences Education, Faculty of Medicine & Health Sciences, McGill University, Montreal Children’s Hospital, Montreal, QC Canada

**Keywords:** Virtual reality, Simulation, Equity, Diversity, Inclusion, Social issues

## Abstract

There is growing recognition that preparing health professionals to work with complex social issues in the delivery of healthcare requires distinct theoretical and pedagogical approaches. Recent literature highlights the significance of employing simulated environments which aim to immerse learners in the experiences of diverse populations and bridge the gap between academic learning and lived realities across a diverse society. Virtual Reality (VR) is gaining traction as a promising pedagogical approach in this context. VR has been argued to offer distinct advantages over traditional educational methods by allowing learners to see the world through the eyes of diverse populations, and to learn about social injustices while immersed in a mediated environment. It also has practical benefits in its capacity to expose large number of students to these topics with relatively modest resources compared to other approaches. This debate article explores VR as an innovative pedagogical approach for facilitating critical reflection, dialogue and transformative learning about social issues in health professions education (HPE). It examines the potential affordances as well as risks and dangers of integrating VR into educational programs and highlights key pedagogical, practical, and ethical considerations. Emphasis is placed on the importance of these considerations in efforts toward ethical, safe, and respectful use of VR in educational settings. This paper contributes to the ongoing dialogue on VR simulation as an innovative approach to HPE and highlights the importance of creating conditions that maximize its educational benefits and minimize potential harms.

## Background

Virtual Reality (VR) can be a potentially transformative pedagogical approach for learning about social issues and promoting critical reflection, dialogue, and transformative learning. It offers a unique opportunity to enhance experiential learning in ways that traditional methods cannot fully achieve. While simulation-based education (SBE) has been shown to be an effective educational modality for teaching technical and non-technical skills in health professions education (HPE) [1, 2], VR extends this potential by enabling immersive experiences that are challenging to replicate in clinical settings. This technology has demonstrated its potential for teaching cognitive and technical skills [3, 4], with a growing emphasis on fostering empathy [5, 6]. However, an underrepresented area of application has been to consider VR as a potentially transformative pedagogical approach for learning about social issues that impact health outcomes. VR can provide learners with a first-person perspective or allow them to witness social inequities while immersed in a mediated environment [7]. The incorporation of technologies such as VR simulation have been argued to hold promise for enhancing experiential learning oriented toward social issues [8–10], suggesting its potential as an innovative pedagogical approach deserving of attention.

The possibilities of VR for raising awareness about complex global challenges gained significant attention following a 2015 TED talk by Chris Milk [11]. In his talk, Milk praised VR as the “ultimate empathy machine,” referring to the VR project he co-created for the United Nations Virtual Reality (UNVR) initiative entitled “Clouds over Sidra.” This project immerses users in the Za’atari refugee camp in Jordan, where Sidra, a 12-year-old girl, shares her daily life, hopes, and dreams, providing a glimpse into the experiences of Syrians living in these camps [12].

The “Clouds over Sidra” VR documentary exceeded expectations, raising $3.8 billion directly from viewers, over 70% more than projected [13, 14]. While this moment propelled VR into the mainstream media spotlight, it built on decades of research in cognitive and behavioral science that explored the medium as a tool to generate empathy and raise awareness on social vulnerability, prompting the United Nations to use it as a tool to support their sustainable development goals.

This rise in prominence also generated a fair amount of scrutiny and sparked debates about the affordances of VR experiences to immerse individuals in another person’s perspective or expose them to instances of injustice, versus concerns about the potential to turn profound human experiences into commercial products that exploit those experiences for profit. These tensions have led to scholarly critiques such as “Rage against the empathy machine” [15], or concerns about VR promoting ‘‘identity tourism for the privileged’’ [16], pointing to the perils of unintentional consequences when the medium’s misuse and goals are misrepresented.

In this debate article, we discuss VR as a pedagogical approach for learning about social issues that impact health outcomes. We examine both the benefits and potential pitfalls of integrating VR into HPE programs and explore the conditions under which it can be used effectively. However, we also emphasize the dangers and consider that its promise may only be fulfilled when pedagogical, practical, and ethical considerations are considered in its implementation. In addition, we propose that users of VR take steps to prevent identity tourism and tokenism, ensure student safety, and foster respect within the learning environment.

## Virtual reality as a pedagogical approach to education about social issues in health professions education

VR simulations offer promising avenues for experiential learning focused on learning about social issues [7–10]. VR has been used to facilitate simulations of intergroup interactions, provide bystander perspectives on discriminatory behaviors, and enable participants to experience these situations from diverse viewpoints [17]. The success of VR in fostering empathy and understanding across different social contexts has been documented and attributed to concepts like presence, immersion, and embodiment in mediated environments [7, 8]. VR enables users to feel situated in three-dimensional spaces, interact with virtual environments, and embody different perspectives. When implemented effectively, VR can create virtual experiences that people perceive and respond to as real [7]. This sense of “being there,” along with the spatial engagement and sensory feedback, has been reported to create a multi-dimensional experience that enhances cognitive processing and information retention [18], and has shown increased engagement and skill acquisition in education and training across cognitive, psychomotor, and affective domains [19].

For instance, instead of simply discussing planetary health and the unprecedented levels of carbon dioxide (CO_2_) in our atmosphere—now 50% higher than pre-Industrial Revolution levels [20]—we can adopt a more immersive approach like Stanford’s Ocean Acidification VR Experience [21]. This VR simulation transports learners to underwater volcanic vents off Ischia, Italy, illustrating the impact of ocean acidification on marine life and highlighting the global effects of CO_2_ emissions on ecosystems. VR has been shown to offer a valuable educational approach to teach about environmental conservation by creating opportunities for learners to visualize complex ecological issues such as deforestation and climate change [22–24]. Markowitz et al. (2018) conducted experiments involving 270 participants, demonstrating VR’s effectiveness in enhancing knowledge and promoting positive attitudes towards environmental issues through immersive experiences like these coastal simulations [24]. The more participants explored the coast and discovered marine objects, the greater the changes in knowledge, suggesting that spatial engagement through VR can enhance learning.

Further, often referred to as the Proteus Effect, an individual’s behavior and attitudes have been reported to be influenced by the characteristics of their avatars in the virtual environment, affecting real-world behaviors [25]. Studies have reported that embodying avatars from diverse backgrounds can reduce implicit biases; for instance, Peck et al. (2013) [26] and Banakou et al. (2016) [27] found that virtual embodiment in different racial avatars decreased implicit racial bias compared to those who used light-skinned avatars. Yee and Bailenson (2007) also observed that negative stereotyping of the elderly significantly decreased when participants were embodied in avatars representing older people [25].

Herrera et al. (2018) conducted a large-scale comparison of traditional versus VR perspective-taking on homelessness, measuring empathy and prosocial behaviors like signing petitions for affordable housing [28]. In Study 1, they compared a traditional narrative-based perspective-taking task with a VR perspective-taking task over eight weeks. Participants in the VR condition reported similar levels of empathy but had more positive, longer-lasting attitudes toward people experiencing homelessness and were more likely to sign a petition. Study 2 compared traditional narrative-based, desktop computer, and VR perspective-taking. All forms increased empathy towards people experiencing homelessness, but significantly more participants in the VR condition signed petitions for social housing compared to other conditions.

Kalyanaraman et al. (2010) assessed VR’s influence on empathy and stereotypical responses towards individuals with schizophrenia [29]. Participants were exposed to different conditions: VR simulation alone, a written empathy reflection exercise alone, both combined, and a control condition. The results showed that participants who engaged in both the written perspective-taking exercise and the VR simulation developed more favorable attitudes towards individuals with schizophrenia compared to those who participated in either condition alone.

A number of studies in cognitive and behavioral science on critical topics such as disability [8, 30], implicit racial bias [26, 27, 31],schizophrenia [29], homelessness [28], and environmental conservation and planetary health [23, 24, 32] are yielding promising results in high priority educational areas. Within the realm of HPE, VR is emerging as an innovative pedagogical tool with potential for educating learners across a range of social issues [33–37]. Early empirical studies, like that of Roswell et al. (2020), have demonstrated VR’s potential to enhance empathy and reduce racial implicit bias among healthcare professionals [34]. Their study with 112 health system leaders, faculty, and staff in a professional development program reported on a VR-based intervention focused on bias and racism. Their findings reported that the majority of participants found VR an effective tool for increasing empathy (94.7%), increasing their own empathy for racial minorities (85.5%), and for changing their approaches to communication (67.1%).

Further research is needed to explore its full potential and to integrate VR effectively into educational frameworks, particularly within HPE contexts.

## VR for transformative learning in health professions education: pedagogical, practical, and ethical considerations

VR, with its unique ability to immerse users in multiple perspectives, presents significant opportunities to facilitate critical reflection and dialogue, and to inform transformative learning about social issues in HPE. These can be facilitated in safe, confidential spaces outside of clinical settings, which are essential for teaching about social awareness and community connection [38]. Learners may develop new or revised interpretations that guide awareness and action towards addressing disruptive and harmful systems [39, 40]. The following discussion examines the range of pedagogical, practical, and ethical considerations for applying VR to teaching about social issues in HPE, summarized in Table 1: Summary of the Pedagogical, Practical, and Ethical Considerations on the Use of VR for Transformative Learning in HPE. It explores the opportunities and drawbacks, drawing from the literature, as well as our experiences and reflections on its uses and possible misuses in SBE.

**Table 1:** Summary of the pedagogical, practical, and ethical considerations on the use of VR for transformative learning in HPE

Considerations	Opportunities	Drawbacks
**Pedagogical**	• Educational approach for transformative learning with unique capabilities in teaching social determinants of health and principles of equity; • Engages learners in unique scenarios (e.g., witnessing social injustices firsthand, experiencing homelessness) to create dissonance and prompt critical reflection.	• Risks of oversimplification and misrepresentation of lived experiences in a single VR scenario, potentially perpetuating misconceptions and biases, while failing to capture the diversity of human experiences; • Potential for adverse events if used without critical reflection and dialogue; • Inadequacy for self-directed learning, as dialogue with peers and facilitator(s) is necessary for transformative learning; • Risk of overlooking potentially more effective educational methods.
**Practical**	• Portable and scalable approach to transformative education that extends beyond traditional SBE settings; • Range of scenarios and topics can be selected on demand; • Viewing can be done in private, enabling learners to process emotions and reactions before the standard debriefing; • Global initiatives provide low-cost to free VR experiences to enhance education on global challenges.	• High fixed development costs necessitate large-scale adoption to achieve cost savings. Without widespread use, there is a risk of misallocating funds and potentially widening the digital divide; • Prioritizing of novelty over educational value may occur.
**Ethical**	• Potential to foster critical reflection on issues of equity, diversity, and inclusion; • Promotes perspectives otherwise unattainable; • Promotes recognition of the situatedness and diversity of human experiences; • Can be used to trouble stereotypical interpretations of experiences or the objectification of the ‘Other’ • Fosters critical reflection and dialogue which may inform transformative learning.	• Without careful implementation and a focus on prompting critical reflection and dialogue with skilled facilitators and peers, VR risks becoming ‘‘identity tourism for the privileged’’ with the potential for miseducation and misrepresentation of human suffering in learners.

### Pedagogical considerations

Leading scholars have argued that promoting social justice and equity in HPE necessitates distinct theoretical and pedagogical perspectives designed to instigate both individual and societal transformation [41–43]. Recent literature and scoping reviews on teaching for social justice and transformative learning have delved into expanding educational endeavors beyond conventional classroom settings, to embrace experiential methods like simulated environments and community-based teaching [44–48]. This approach aims to immerse learners in the authentic experiences of communities, bridging the gap between academic learning and the lived realities present across a diverse society.

Aligned with transformative learning theories, VR may be fruitful for learning about the social determinants of health and principles of equity, and as a means to foster critical reflection in HPE. Transformative learning begins with a catalyzing event referred to as a disorienting dilemma, which disrupts habitual and often unconscious frames of reference [39, 40]. This state of confusion or uncertainty triggers critical reflection on deeply ingrained assumptions, challenging the previously held, taken-for-granted interpretations. It prompts learners to take a reflective stance on assumptions, beliefs, and values, and evaluate the influence of social structures and institutions that create or sustain disadvantage [39, 40, 43, 49]. This process fosters rational discourse and communicative learning, where meaning is discussed, negotiated, or validated with others, frequently involving a teacher or mentor. Successfully navigating this process leads to perspective transformation, influencing one’s worldview and capacity to act as change agents through a series of transformative steps.

VR can trigger such disorienting dilemmas by offering unique experiences unattainable through other educational modalities, providing a lens through which to discuss social and health equity issues that trainees may not otherwise encounter. Bailenson’s DICE framework (Dangerous, Impossible, Counter-productive, and Expensive) identifies where VR offers unique educational benefits [7]. For example, VR can simulate impossible scenarios, such as witnessing social injustices firsthand, travelling through time to see a person’s life story, experiencing repeated discrimination, or living in a situation of homelessness. This can create dissonance and prompt critical reflection on learners’ perceptions, beliefs, values, and assumptions, potentially leading to a shift in perspective.

At the same time, it is important to acknowledge that there are complexities and risks in simulating human experiences. Questions arise about whether brief immersive experiences can authentically capture the complexity of lived experiences, raising concerns about the potential tokenization, oversimplification, and selective portrayal of an identity or experience. Moreover, these experiences can misrepresent individuals and cultures through inaccurate depictions, and risk perpetuating misconceptions and reinforcing biases and stereotypes rather than challenging them [50]. These concerns apply to VR and other forms of simulation. Silverman (2015) conducted a study to determine whether simulating the experience of blindness with a blindfold or interacting with individuals who are blind leads to greater empathy and reduced stereotyping [51]. While both methods increased empathy, participants who interacted directly with individuals who shared the lived experience showed greater long-term empathy. While resources generally inform the pedagogical approaches we use, it is also crucial to evaluate whether another well-designed educational activity, developed in consultation with the diverse voices of those with lived experience, may be better able to achieve educational goals.

As we explore technological innovations in SBE, we need to also critically examine the risks and dangers. For instance, VR, often praised as a self-directed learning tool, can be problematic when it comes to solo experiences of critical reflection and transformative learning. Critical reflection and transformative learning require dialogue and opportunities for shared meaning-making with facilitators and peers in an environment of trust [40]. A safe environment to discuss, negotiate, and validate problematic assertions or presuppositions is required. Engaging in VR learning activities alone, without skilled facilitators and a safe environment for processing critical reflections, may hinder the learning process, with the risk of negative associations going unaddressed.

Further, the absence of opportunities to debrief VR experiences can have adverse outcomes. For example, a 2010 study by Kalyanaraman et al. investigated VR’s impact on empathy towards individuals with schizophrenia [29]. Participants who received the VR simulation with a written reflection exercise written empathy condition demonstrated greater empathy and more positive perceptions. In contrast, those who did not receive the written reflection with the VR simulation expressed a greater desire for social distance. This highlights the caution needed regarding the potential adverse events of using VR without a reflective educational component.

### Practical considerations

VR simulation offers practical applications. The significant reduction in the cost of VR headsets over the last decade has increased accessibility, particularly when compared to other simulation-based technologies that require greater investment, resources, and infrastructure [52]. While the initial investment in VR technology may seem significant compared to hiring a simulated patient to perform a scenario, the long-term value is substantial given the scalability of the technology, and its capacity to accommodate large numbers of learners. Additionally, a range of scenarios and topics can be curated on demand to meet the evolving needs of learners and curricula. The potential for widespread adoption and portability of VR extends the boundaries of SBE beyond traditional walls and opens new avenues for delivering simulation across education and healthcare systems. Moreover, learners can participate in the VR experience in private, and process emotions or reactions prior to the standard debriefing, where a skilled facilitator and peers foster critical reflection and dialogue about the learning and insights gained during the simulation.

However, assessing the practical benefits of VR initiatives raises critical questions. Initiatives such as “VR for Good” and “VR for Impact,” supported by leading VR head-mounted display (HMD) companies such as Meta and HTC Vive, have committed millions of dollars to the development of VR experiences aimed at raising awareness and improving education about global challenges [53–55]. These experiences have employed VR documentary filmmakers to create immersive experiences for widespread distribution, many for free or at modest cost. However, if VR HMDs are not widely available, limiting who can access and benefit from these experiences, it raises questions about the return on these investments, and whether these funds could be more effectively directed to other educational priorities, or invested in participatory partnerships for the co-creation of programs and services with underserved communities. In addition, there are further issues of equity to consider. VR requires access to HMDs, reliable internet, and technical support, which could widen digital divides by being inaccessible to large segments of society [56].

While VR holds significant potential for initiating transformative learning, it can also be a seductive tool that attracts learners because of its novelty. Termed the “technological novelty effect,” this allure can boost interest but may prevent a clear assessment of true educational value [57, 58]. This initial fascination can overshadow established teaching methods and instructional design principles better suited to learning objectives. Radianti et al.’s (2020) systematic review of immersive VR in higher education reported a lack of learning theories to inform VR application design, with an overemphasis on usability over learning outcomes [56]. Thus, educational experiences need to be carefully selected based on identified needs and learning objectives outlined in the curriculum analysis, ensuring the use of pedagogically sound theories and approaches to achieve intended goals, rather than simply using VR as a novel delivery medium.

### Ethical considerations

While there is a growing consensus on the use of critical pedagogies and transformative learning in the development of educational programs for social justice and equity [38, 41–43], much remains to be discovered about the most effective educational modalities for achieving these goals [59]. Virtual simulations are recognized for integrating diverse perspectives and backgrounds, enabling the development of more inclusive pedagogies that prepare learners to meet the diverse needs of the communities they serve [60–63]. VR, in particular, offers the added benefit of reducing users’ psychological distance from a phenomenon by allowing learners to step into simulations of others’ experiences and gain perspectives that would otherwise be inaccessible to them. It has also been shown to have a more pronounced impact on influencing social attitudes than non-immersive interventions [9].

Despite VR’s potential to promote diversity and inclusivity, critics have highlighted the risk of it becoming “identity tourism for the privileged” [15], and of it creating a misleading narrative that suggests human suffering can be understood through brief engagement with a social issue. This perspective fails to capture the depth and complexity of what it means to experience social marginalization. Nakamura argues that this type of VR can turn empathy into a commodity, curated and licensed for viewing, potentially objectifying the Other [16]. Others argue that the framing of these VR experiences can be ethically flawed, suggesting that a brief encounter with a simulated reality cannot represent the lived realities of complex human experiences [15, 50, 64, 65]. Finally, mainstream attention has focused heavily on VR as the “ultimate empathy machine,” with many research studies in the area focusing primarily on VR-induced empathy and perspective taking [7, 66]. They suggest that VR allows one to understand and share the feelings of another through brief simulated experiences [16].

We propose that it is more productive from an ethical perspective to position VR as an educational approach to learn about social issues and to prompt critical reflection rather than as an all-encompassing means to understand or empathize with a lived experience. This is similar to how we use simulated patients to enact a scenario, knowing that it does not fully encompass the reality of each patient. This underlines the importance of skilled facilitators: to prebrief and debrief with learners toward recognition of the situatedness and diversity of human experiences; trouble stereotypical interpretations of experience or the objectifying of the ‘Other’ that may emerge; and foster a learning environment attuned to the dimensions of ethically sound VR experiences.

## Stimulating a curricular path forward through VR simulations

As educators, we have drawn on our experience in VR simulation, and the literature, to explore the promise and risks of using VR as an approach to transformative learning in HPE. Our debate balances VR’s promise with its potential drawbacks and proposes pedagogical, practical, and ethical considerations for the responsible use of this technology. While our conclusions support and applaud the promise of VR in this context, we advocate for strategies to mitigate risks, and judicious educational design choices, to minimize unintended consequences.

Considering the emotionally charged nature of these issues, especially for those who identify with them, it is important to bring careful attention to the learning environment. Providing opportunities for privacy during VR viewing and allowing students to opt-out if they find the content triggering can foster engagement and create a safe learning space. Post-simulation reflexivity should be incorporated for all stakeholders, including faculty debriefers and the simulation team, as a means for reflection on what went well, potential harms and biases that may have emerged, and consideration of how these could be mitigated in the future [67]. This approach ensures continuous learning and integration of best practices into educational sessions.

In addition, despite the novelty of the technology, the curriculum design should guide the development of learning activities in alignment with best practices. The International Nursing Association for Clinical Simulation and Learning (INASCL) Standards of Best Practice provide a framework for the effective integration and use of simulation-based education, with guidelines for simulation design, facilitation, prebriefing and debriefing, among other aspects [68, 69]. The prebriefing prepares learners by outlining the objectives and scope of the educational intervention, providing an overview of the simulation experience, and establishing psychological safety for engaging with the content [70]. It allows for the opportunity to clarify the goals of the activity, ensuring that the learners understand that the simulated scenario does not fully represent the lived realities of diverse identities and experiences. It emphasizes that the VR simulation offers only a glimpse into a complex social issue rather than encompassing the experience of all individuals. The debriefing then facilitates critical reflection and dialogue about the VR simulation, allowing learners to process emotions, reactions, and perceptions [71] while creating spaces for discussions about social determinants of health and principles of equity in the delivery of care. Clearly defining the goals and limitations of the activity, and facilitating critical reflection and dialogue on the simulations insights, will help safeguard against “identity tourism” and establish VR as an educational tool.

Finally, engaging individuals with first-hand lived experience in the co-design of VR educational activities can promote a more inclusive and authentic representation and enrich the quality of the educational design. While acknowledging the challenge of obtaining representative voices, particularly since the most pressing perspectives are often absent from our healthcare and academic systems [63, 72–74], it is beneficial to involve someone with the lived experience of the topic. These first-hand perspectives can offer valuable insights into potentially stereotypical or offensive approaches, helping to identify and mitigate the risks of misrepresentation, stigmatization, and bias to create supportive learning environments. Active involvement of these individuals encourages their participation in the educational design process and integrates diverse perspectives and expertise through a co-development model. In reviewing VR modalities, investigations into “who” was involved in the design process are also warranted. Such approaches can foster inclusive, engaging, and accessible educational experiences for participants, and help to promote ownership and collaboration.

## Conclusion

With the pressing need to design better performing health systems, it is imperative to prepare healthcare professionals who possess not only clinical competence but also the ability to comprehend and address the broader social determinants impacting health outcomes. To equip practitioners and learners to respond to the intricate health needs of the communities they serve, innovative approaches are essential.

We contend that VR, with its unique ability to immerse learners into diverse, on-demand scenarios, and foster engagement with a range of social issues, offers a novel educational experience. This first-hand, immersive exposure, when well designed, presents significant opportunities for learners to engage in critical reflection and dialogue essential for transformative approaches to learning. However, it is also important to acknowledge the challenges. Serious critiques about the dangers of identity tourism, tokenism, the misrepresentation of complex human experiences, and more highlight the importance of ongoing reflexive attention into how these approaches are implemented.

While the integration of technology such as VR has the potential to enhance learning, and to promote a more inclusive and compassionate approach to care, the risks must be thoughtfully weighed and considered in any implementation efforts. While there is well-established evidence of VR’s potential to foster learning about social issues across a range of disciplines, research into its role in preparing future health professionals to work effectively with historically underserved populations is limited. Further research is needed to assess its effectiveness and applicability and the conditions under which it may support or hinder teaching and learning about social issues in HPE. Striking a balance between implementation goals and pedagogical, practical, and ethical considerations is critical as we delve deeper into the potential of VR in HPE. Caution is warranted in the judicious use of this pedagogical innovation, with principles of respect and integrity considered within the design of safe learning environments.

## Data Availability

Not applicable as no datasets were generated for this article.

## Abbreviations

CO_2_: Carbon dioxide
DICE: Dangerous, impossible, counter-productive and expensive
HMD: Head-mounted display
HPE: Health professions education
INASCL: International Nursing Association for Clinical Simulation and Learning
SBE: Simulation-based education
VR: Virtual Reality
